# Dental Abstracts

**Published:** 1854-04

**Authors:** 


### Dental Abstracts.

*Another European Principle Americanized.*—In the October No. of American Journal of Dental Science, for 1853, we have an article under the above caption by Wm. M. Hunter, M. D., of Cincinnati, Ohio, which we regard as of some practical importance to the Profession. There are many cases, we are satisfied, where artificial teeth can be thus supplied, with much less injury to the other teeth and far more comfort to the patient, than by the means of clasps. We have tried the principle enough to satisfy us, that it is far more pleasant in the mouth, and retains the artificial teeth with equal if not more firmness.

We should have been glad to give two or three drawings of cases which accompany the article, but have not the cuts, and the description is sufficiently accurate to enable any good Dentist to accomplish the same without difficulty. The Doctor remarks that,

In the year eighteen hundred and fifty-one, a gentleman of Cincinnati, during a visit to Europe, employed a Parisian dentist to supply the two inferior bicuspid teeth of the right side, together with the left anterior bicuspid of the same jaw, which operation was performed by carving the teeth and the plate (so to speak), very neatly from the bone of the hippopotamus. The fit was admirable, and the finish unexceptionable; the novelty, however, of the work consisted in the method of retaining it in the mouth, which was as follows: After the piece was fitted, four holes were drilled into the bone on the sides approximating to the natural teeth at a point opposite their necks, into which were inserted cylinders of hard wood, protruding just sufficiently to produce the amount of pressure necessary to retain it in place; unfortunately, however, the natural teeth were separated, and the continual pressure of each new plug rendered necessary by the yielding of the teeth, caused an opening that the artificial teeth would no longer fill, and another contrivance became necessary.

Seeing the principle, and the evil results of its application in this case, it required but very little thought to overcome the difficulty, and the consequence is, an application of the principle to gold plates with entire success, by means of tubes soldered to the plate at proper points and filled with compressed wood.

The advantages in many cases must be apparent to the thinking dentist, but perhaps it might not be amiss to enumerate a few.

The fixture is held in place with greater firmness than by means of clasps.

In some instances where I have used clasps, I have also used the tube in combination to give stability for masticating purposes.

The injury to the natural teeth must be much less, owing to the smaller amount of surface in contact.

If decay should take place, it would require but an ordinary filling to restore the tooth.

It prevents that peculiarly disagreeable sensation experienced, particularly in fruit season, upon removing and replacing artificial teeth.

After having tested it for more than a year, I am satisfied that it greatly lessens the chance of decay in those cases where it can be applied, and I have removed the clasps in some old cases with great satisfaction to my patients.

My method of applying the tubes is as follows: After swaging, the plate, as usual, is tried in the mouth, and an accurate impression of the teeth to be used, is taken over the plate, as recommended by Dr. Arthur, in the American Journal, which will show the exact position of the tooth in its relation to the plate; after which the edge of the plate surrounding the teeth to be made use of, should be doubled or wired, when the tubes may be soldered at their proper points, taking care never to apply pressure to one side of a tooth without some means of counteracting the effect—that means being either a *sufficient number* of natural teeth contiguous to the tooth to be used, a counter tube, an arm of metal, or an artificial tooth, must depend entirely upon the nature of the case.

At times, it is well to tube but one side of the plate, and clasp the other; in cases where the crown of the tooth is much larger than the neck, a beautiful application may be thus made.

The tubes should be from one-eighth of an inch down to one line in diameter, and should be filled with whiting before applying heat, to prevent them from filling with solder at the time of soldering to the plate. They should be placed upon the plate so carefully that the mouth of the tube will come in contact with the natural tooth, as it is desirable to have the wood protrude but very slightly beyond the orifice.

Where it can be properly done, the tubes are soldered at the same time the teeth are, as it saves much trouble in fitting; it can not, however, be very well done where it is designed to fit a tooth over a tube, but can very readily be done where the tube is designed to fill the angle caused by the meeting of the stay and plate, in the incisors and canine teeth, and where a canine is used for a bicuspid, building over the tube with metal to form the inner cusp.

The January No. of the Journal, for 1854, contains first an article on "Chemistry of the Metals." The metal under consideration in this number is Platinum, and to direct the attention of the Profession to this metal, and its capabilities of being prepared into *Sponge*, which may answer for filling teeth. The recent introduction of Crystal Gold, for plugging teeth, would seem to indicate that as good, if not a better article could be prepared from Platinum. One known property of this metal is its capability of being welded. We hope that those who are now engaged in manufacturing Crystal Gold, will try their hand at Platinum.

In the January No. of the News Letter, for 1854, we have Dr. T.W. Evans' address on the regulation of teeth continued. We regard this as a very important subject, and have no doubt but that in a few years this kind of practice in the Profession will be quadrupled. We are satisfied that we now have ten cases to treat, of this character, to where we had one ten years since, and we regard the amount of benefit we confer in this way, greater than in any other part of our practice.

Our mechanical fixtures are few and very simple, much more so than those used by Dr. Evans, yet we are not prepared to say they are in all cases as efficient. We shall, however, in the present number give a part of Dr. Evans' article on this subject, and in our next issue give the remainder, hoping by that time to have some drawings prepared illustrative of the mechanical fixtures we use.

Dr. Evans remarks that,

The principal desiderata in the apparatus for the regulation of teeth are:

1st. A firm support which shall not loosen, or in any way injure, the teeth to which it is attached. 2d. A steady and sufficient pressure, which can be graduated to suit particular cases, and particular stages of an operation. 3d. Great delicacy of construction, that the apparatus may be as light as possible, so as neither to injure nor annoy the patient. 4th. Finally, a mechanism as simple as the case will admit of, in order to economize both labor and time.

I am aware, gentlemen, that in all these important respects great progress had been made long anterior to my departure from America. On the other hand, I presume, I am only stating the experience of every member of the profession when I say not only that the apparatus used at that period was far from being perfect, but that there is much to be desired even now. I infer thus much not only from the nature of the case, and from my own daily observation, but because in reading the valuable Dental journals which come to me from America, I see that more light is called for, and less given, upon this subject than upon almost any other. Doubtless, however, many improvements have been made which have not yet been made public, and I can not help remarking, in this connection, that the delay in publishing the valuable discoveries which are made from time to time in our profession, is deeply to be lamented, for the advance of the Science depends to a great extent upon such discoveries being immediately announced. I may add, that it is possible—probable even—that some of the improvements which have occurred to me in the seclusion of my laboratory, may also have occurred, in the same manner, to some of you. It is not impossible, either, that among the discoveries made here, the knowledge of which has not yet reached the Old World, there may be some far superior to my own. If so, I shall be glad to have them communicated to me, that I may introduce them into European practice.

Case I.—The first case to which I would call your attention, as illustrating the advantages of the apparatus, is that of

*Restoring an oblique upper incisor to its place.*—The first thing, of course, is to make room for the operation, if, as is generally the case, room is required. This is accomplished by bits of India rubber one-sixteenth of an inch in thickness, which are inserted between the irregular tooth and its neighbor on each side, and is prevented from sliding up to the gum by means of floss silk wound tightly round the middle of the tooth. The necessary space obtained, I take a piece of hard-drawn wire, about the size of a common pin, and winding one end of it twice round the middle of the oblique tooth, very closely, I pass the other end along to the bicuspids or molars, to which I attach it by means of a joint—mechanism of two nicely adjusted bands or rings—forming a kind of yoke, which is slipped over two of the bicuspids or molars, and is prevented from slipping up to the gums by means of wire clamps which hook on to their grinding surfaces. These clamps may be so constructed, when an operation requires it, as to prevent the teeth of the upper and lower jaw from coming in contact with, or overlapping each other.

It will be seen at once, that if this apparatus is rightly applied, the hard-drawn wire being elastic, and serving at once as lever and spring, will bring the oblique tooth round to its place in a short time. But in all cases where, from the age of the patient, or for any other cause, the tooth is too refractory to submit to so gentle a lever, I modify the apparatus as follows:

*First.*—Carefully adjusting a gold band (made of 22 carat gold, so as to be sufficiently malleable), round the center of the tooth to be operated upon. On the front of this band, and directly across it, I solder a small gold tube, the bore of which is about the size of a common pin; through this tube I pass a hard drawn wire, fitting very closely, and then attach the outer end of it (which has a loop or eye for the purpose), to the yoke or skeleton-cap above described, which is previously fitted to two of the molar or bicuspid teeth. In this way it will be seen, we have a more powerful lever than before, and one which is equal to any emergency.

The operation being completed, the tooth is left in its place, by means of a small flattened wire, (somewhat smaller than a knitting needle), which I pass in front of the incisors as far as the eye teeth on each side, to which it is attached by means of silk ligatures; this wire band is prevented from slipping up by means of a small frame-work with two hooks, which I solder to it, and which, taking hold of the cutting edge of the central incisors, afford a firm and steady support. It is obvious that the wire band thus arranged and sustained, will easily keep the restored tooth in its place. The length of time it must be worn depends upon circumstances. It should be removed as often as possible, for the purpose of cleansing.

We have also, in the January number of the News Letter for '54, an article on the Nerve Cavity, by J. F. R. Flagg, M. D., D. D. S., Philadelphia. The Dr. gives some good directions in relation to the preparation of the cavity before filling, and we perfectly agree with him in relation to the prospect of success being greater in those cases where we destroy the nerve, "than when nature has performed that operation for us."

In relation to the use of arsenic, the Dr. remarks—

I have upon a former occasion given my views as to the time necessary for arsenious preparations to remain in a tooth —"five hours." It is from very careful observation in this respect, that I mention this period as best calculated to accomplish the desired object. It will be found sufficiently long to paralyze the nerve, and enable us, within twenty-four hours after its application to introduce a broach, and withdraw the nerve or nerves entire; and it does not in that time sufficiently decompose animal fibre, either to hazard the risk of leaving any portion behind, or extend its deadly influence beyond what we desire to.

As an illustration of what a minute quantity of arsenic is capable of doing, I will relate the following extreme case:— Five years since, a lady, about thirty years of age, desired me to extract the six front upper teeth, for the purpose of having them replaced by means of plate-work. Upon examining the case, I found that every root was alive, and unexceptionable, for the porpose of a pivot operation. This I explained to her, and she concluded to adopt this method of practice. Upon reaching each nerve as I progressed in the operation, I plunged in the broach, and so far destroyed it, as to enable me to drill as deep as was required.

I proceeded with my work adjusting, each tooth with a soft wood-pin, until they were all prepared for final setting. At this point, it became too late to allow of me completing the operation, and as there appeared to be some little sensation remaining in all the roots, I concluded to put into each a small quantity of the arsenious preparation; directing her to pick it out in five hours, to rinse the mouth well with cold water, and refill the cavities with either wax or clean bits of cloth. She was to have returned to me the following day, but she did not. It was not till ten days that my patient presented herself. She said she had not been very well but that her teeth had been so comfortable, she did not feel any anxiety about them. On seeing bits of rag in the roots, I inquired if she had put in fresh pieces every day; she said, "Oh, no—they have felt so perfectly easy ever since you fixed them, I have not disturbed them since. *I had "fixed them.*" The first root I touched left the socket with the simple effort to withdraw the pledget, and so with all the rest. Their surrounding membranes were completely destroyed, and I feared, at the time, that the mischief had extended sufficiently to result in extensive sloughing; but by the free use of astringents this evil was avoided. This, of course, led to the necessity of complying with her previous views, and in due time I furnished her with teeth upon the plate.

We have referred in another place (see page 220) to the preparation of Platinum as an article for filling teeth. We give an abstract in relation to the mode of purifying and working this metal, which should have been inserted on page 220. There is much in these articles especially interesting to the Profession. They are from the pen of Prof. R. N. Wright, A. M., M. D., and may be found in two or three of the last numbers of the Dental Journal.

We next proceed to lay before our readers, the method of purifying platinum, and inasmuch as the credit of the best process seems to be almost universally ascribed to Dr. Wollaston, we subjoin his plan, as copied from the Philosophical Transactions of 1829.

"The usual method of giving chemical purity to this metal, by solution in *aqua regia*, and precipitation with *sal-ammoniac*, are known to every chemist; but I doubt whether sufficient care is usually taken, to avoid dissolving the irridium contained in the ore, by due dilution of the solvent. In an account which I gave in the Philosophical Transactions of 1804, of a new metal, *rhodium*, contained in crude platinum, I have mentioned this precaution, but omitted to state to what degree the acids should be diluted. I now, therefore, recommend, that to every measure of the strongest muriatic acid employed, there be added an equal measure of water; and, that the nitric acid used, be what is called '*single aquafortis*;' as well for the sake of obtaining a purer result, as of economy in the purchase of nitric acid. With regard to the proportions in which the acids are to be used, I may say, in round numbers, that *muriatic acid*, equivalent to 150 marble, and *nitric acid*, equivalent to 40 marble, will take 100 of crude platinum; but, in order to avoid waste, and render the solution purer, there should be in the menstruum, a redundance of 20 per cent, at least, of the ore. The acids should be allowed to digest three or four days, with heat gradually raised. The solution being then poured off, should stand till a quantity of fine pulverulent ore of *irridium*, suspended in the liquid, has subsided; and should then be mixed with 41 parts of sal ammoniac, dissolved in about five times their weight in water. The first precipitate which will thus be obtained, will weigh about 165 parts, and will yield about 66 parts of pure platinum. As the mother liquor will still contain about 11 parts of platinum, these, with some of the other metals, yet held in solution, are to be recovered by precipitation from the liquor, with clean bars of iron, and the precipitate is to be redissolved in a proportionate quantity of *aqua regia*, similar in its composition to that above directed; but in this case, before adding *sal-ammoniac*, about 1 part by measure of strong muriatic acid should be mixed with 32 parts by measure, of the *nitro-muriatic* solution, to prevent any precipitation of palladium, or lead, along with the ammonia-muriate of platinum.

"The yellow precipitate must be well washed, in order to free it from the various impurities, which are known to be contained in the complicated ore in question; and must ultimately be well pressed, in order to remove the last remnant of the washings. It is next to be heated with the utmost caution in a black-lead pot, with so low a heat as just to expel the sal-ammoniac, and to occasion the particles of platinum to cohere as little as possible; for on this depends the ultimate ductility of the product.

"The gray product of the platinum, when turned out of the crucible, if prepared with due caution, will be found highly coherent, and must then be rubbed between the hands of the operator, in order to procure, by the gentlest means, as much as can possibly be so obtained, of metallic powder, so fine as to pass through a fine lawn sieve. The coarser parts are then to be ground in a wooden bowl, with a wooden pestal, but on no account with any harder material, capable of burnishing the particles of platinum; since every degree of burnishing will prevent the particles from cohering in the further stages of the process. Since the whole will require to be well washed in clean water, the operator, in the later stages of grinding will find his work facilitated by the addition of water, in order to remove the finer portions, as soon as they are sufficiently reduced, to be suspended in it."

We have, in the same number of the Journal, an article on Mechanical Dentistry, by John Lewis, D. D. S., of Buffalo, New York.

After noticing the condition of the mouth necessary for the reception of artificial teeth, he first describes the method of taking an impression; for this purpose he prefers wax, recommending as essential to success in this part of the operation, cups made to suit models of the mouth ; these are oiled before the wax is put in them.

We can not but regard, however, that the Dr. errs when thinking that wax, prepared with even the care he takes, will give as good an impression as plaster.

The Dr. recommends the following as the best alloy for gold plate to be used for dental purposes : Take 1 gr. of copper, 2 grs. of silver, and 24 grs. of American gold ; or take the three-cent pieces, which contain about the relative proportions of copper and silver.

After having adjusted the plate to the mouth, and fitted a wire border to the same, the next part of the operation—and one which the Dr. regards as an important improvement in the mode of inserting artificial teeth—is, to arrange the standards to which the teeth are to be attached.

These are arranged to the base-plate in three sections : first, a piece of plate long enough to form the circle for the six front teeth, and in width sufficiently wide to suit the length of the teeth to be used. This is first attached to the plate. The mode of doing this, and the side standards, we give in the Dr.'s language, merely remarking, that when we first located in Cincinnati, Dr. Cook pursued much the same method, and we had several arguments with him, on what we regarded then as the uncertainty and tediousness of the operation, as compared with the present general plan, which was then somewhat new. The Dr. remarks that—

The operator should carefully observe, while taking the impression and trying the plate, the position and prominence of the gums, and each place on the plate it is necessary to set the teeth. By practice, and careful attention, he can, in this way, ascertain by the eye the proper place on the plate for the arrangement of the standard, which will seldom be incorrect. The standard should now be placed in the required position, and securely bound there with small annealed iron wire; it can then be barely attached on to the plate, with a small piece of solder, when the wire should be removed, and the plate again tried into the mouth, to ascertain if it is in the position required.

If there are natural teeth in the lower jaw, these will determine the position, as the standard should shut over the lower teeth, when the jaws come together in the natural way; but if there are no front teeth in the inferior maxillary, its place should be determined by the relative position of the two jaws, taking into account the restoration of the contour of the face and lips, making suitable allowance for the thickness of the teeth on the outside of the standard. If the standard is found to be too far out, or too far in, or in any way different from what it should be, it should now be broken from the plate with a pair of pliers, or if it so firmly attached as to endanger the plate, it can be placed upon the charcoal, and the solder melted, when it can be removed with a slight blow with the end of the blow-pipe. The standard should now be re-adjusted, and fastened as before, with solder. The plate should then be again put into the mouth, and the standard having been adjusted to it in its right position, it should be trimmed to the required length. The median line or center should now be marked on it with a sharp instrument, to designate the proper place for the central incisors.

The plate may now be removed from the mouth, and a notch filed in the standard at the point thus marked, so that if the former should be obliterated in heating the plate, no mistake will be made in adjusting the teeth. The standard should now be firmly bound in its place with iron wire, to prevent the liability of its being moved, and then permanently soldred to the plate.

*Soldering the Standard to the Plate.*—The standard having been properly adjusted, borax ground with water should be placed along the line of union, and this can be done best with a camel's-hair pencil. It should be of the consistence of cream, and applied wherever it is desired the solder should flow. If a sufficient quantity is not applied, the solder will not flow freely, but form itself into a globule where it is placed, or will simply adhere to the body of the plate. No more solder should be employed than is necessary to unite the backing securely to the plate.

The standard having been filed down to the length required for the teeth, the latter may be selected, taking care to have them correspond in shape with the natural organs, and the complexion, and general contour of the face as nearly as possible. If they do not do this, they may disfigure, instead of enhancing the beauty of the patient.

The teeth are adjusted to the standard, beginning with the central incisors, by punching holes in it to correspond with the rivets in their inner or palatine surface, and for this purpose a common dentists' punch is used; but it will require some practice to make them in the proper place. The best manner of doing it is to place the tooth upon the plate and standard, as near in the position it should be as possible, and then press it firmly against the latter. This will leave slight marks indicating the place for the rivets. Slight indentations may now be made at the places thus marked with the punch, and the tooth again tried to ascertain if they are in the right place; if so, they can now be punched through, and the inside counter-sunk, so as to form a place in which to make a head in the rivet. To do this, a suitable instrument may be nsed.

In adjusting the central incisors, a very narrow space may be left between them, or their approximal surfaces may barely touch, but care must be taken that they do not crowd each other, and the base of the teeth should be ground until they fit the plate.

The lateral incisors should next be adjusted, and these should be a very little shorter than the centrals, and the cuspids slightly longer, about half the length of the cusp on the end.

But as yet the teeth are not fastened, the holes only having been punched and the teeth fitted. Each one, however, will keep its proper position, if the holes are not made too large, a thing which should be carefully avoided. If this is neglected, the teeth cannot be securely fastened to the standard. The incisor and cuspid teeth having been accurately fitted, the ends of the standard, back of the last mentioned teeth, should be filed off.

The same No. of the Journal contains an article on the essential oil of tobacco, as a remedy for the tooth-ache, (giving it as a German remedy), by J. L. Levison, D. D. S.

The remedy appears to be so powerful, that Dr. Levison has abandoned its use.

We give the mode of preparing it, and also his last case. He says:

I procured a clay-pipe, filled it with tobacco, and on the extremity of the tube a mouth-piece was put, cut from the lower end of a goose-quill, and into this tube or mouth-piece I inserted a portion of white cotton wool. The tobacco was then ignited, and a few streams of smoke were drawn through the cotton into the mouth, and from the latter, sent in graceful curls into the circumambient air. A few seconds sufficed to saturate the cotton with a dark yellowish oil.

Case 3.—My last case was likely to be attended with some personal danger. A very tall and powerful farmer came into a shop in which I was, giving some orders, when he said, "I am mad with the tooth-ache." The shopman, who knew me, answered, "well this gentleman can cure you." "Ah!" sighed the yeoman, "but not without taking out the tooth, and I had my jaw nearly broken, when a doctor tried it some time since." I proposed the German remedy, and retired into a back room to prepare it. There my stalwart patient came on being summoned. The cure was instantaneous, and I could have exclaimed with Cæsar, "*veni, vidi, vici*;" for with a regular halloo, he shouted, "the pain's gone! the pain's gone!" And as I did this, *sans* a fee, I left immediately afterward, particularly as the robust young gentleman seemed quite well. Soon afterward he was taken very sick and dizzy, was conveyed to the inn, and had to go to bed. When he recovered, he sought for me everywhere, and threatened "to give me a sound drubbing;" so as this sort of practice was profitless, I have abandoned it ever since.

